# Decreased risk of liver and intrahepatic cancer in non-*H. pylori*-infected perforated peptic ulcer patients with truncal vagotomy: a nationwide study

**DOI:** 10.1038/s41598-021-95142-z

**Published:** 2021-08-02

**Authors:** Shih-Chi Wu, Han-Tsung Cheng, Yu-Chun Wang, Chia-Wei Tzeng, Chia-Hao Hsu, Chih-Hsin Muo

**Affiliations:** 1 School of Medicine, China Medical University, Taichung, Taiwan; 2 Trauma and Emergency Center, China Medical University Hospital, No. 2 Yuh-Der Road, Taichung, 404 Taiwan; 3 Department of Trauma and Emergency Surgery, China Medical University Hospital, Taichung, Taiwan; 4 Management Office for Health Data, China Medical University and Hospital, Taichung, Taiwan

**Keywords:** Cancer, Diseases, Medical research, Oncology, Risk factors

## Abstract

The vagal nervous system is central to the physiological responses and systemic diseases of the liver. We evaluated the subsequent risk of liver and intrahepatic cancer (HCC/ICC) in non-*H. pylori* (HP)-infected perforated peptic ulcer (PPU) patients with and without vagotomy. Hospitalized PPU patients who underwent simple closure or truncal vagotomy/pyloroplasty (TVP) in the National Health Insurance Research Database from 2000 to 2008 were enrolled. The exclusion criteria included: (1) Multiple surgeries for PPU were received at the same admission; (2) Any cancer history; (3) Previous peptic ulcer-associated surgery; (4) HP infection history; (5) Viral hepatitis infection history; (6) Follow-up duration < 1 year; and (7) Age < 18 years. The risks of developing HCC/ICC in PPU patients with and without vagotomy were assessed at the end of 2013. To balance the baseline condition between groups, we used the propensity score matched method to select study subjects. Cox proportional hazard regression was used to estimate the hazard ratio and 95% confidence interval (CI) of HCC/ICC. Before propensity score matching, 675 simple suture patients and 54 TVP patients had HCC/ICC, which corresponded to incidences of 2.11 and 0.88 per 1000 person-years, respectively. After propensity score matching, 145 simple suture patients and 54 TVP patients experienced HCC/ICC, which corresponded to incidences of 1.45 and 0.88 per 1000 person-years, respectively. The TVP patients had a 0.71 (95% CI 0.54–0.95)- and 0.69 (95% CI 0.49–0.97)-fold risk of developing HCC/ICC compared to simple suture patients before and after propensity score matching. Our findings reported that, in the Asian population, TVP decreases the risk of HCC/ICC in non-HP-infected PPU patients compared to simple closure patients. However, further studies are warranted.

## Introduction

Liver cancer is the fifth most common cancer and accounted for 9.1% of all cancer deaths worldwide in 2012^1^; it became the fourth most common cause of cancer-related death worldwide in 2019^2^. Most liver cancer cases are noted in low- and middle-resource countries, particularly in Eastern Asia, and remain an important global health issue^2^.

The majority of primary liver cancers (75–90%) are hepatocellular carcinomas (HCCs), which are malignant tumors of liver parenchymal cells. The other primary liver cancer is intrahepatic cholangiocarcinoma (ICC), a tumor of intrahepatic ductal lining cells^3^. Common risk factors for HCC include hepatitis B virus (HBV) and hepatitis C virus (HCV) infection, obesity, diabetes, fatty liver disease, aflatoxin, alcoholic cirrhosis, smoking, and iron overload^4^, while risk factors for ICC include chemical exposure, parasites, sclerosing cholangitis, viral hepatitis, and cirrhosis^5^.

On the other hand, the vagus nerve and the inflammatory reflex are essential for immunity and metabolic homeostasis^6^. Anatomically, the hepatic parasympathetic nerves branch off the vagus nerve and innervate the liver^7,8^. It has been reported that increased vagal parasympathetic tone is associated with peptic ulcer diseases in humans^9,10^ and related to gastric stress ulceration development in rodent studies^11^. In addition, studies have shown an association between the hepatic nervous system and clinical diseases, such as type 2 diabetes mellitus^12,13^, obesity^14^, and cirrhosis^15^.

We hypothesized that the severance of the vagus nerve (vagotomy) would deinnervate the liver, resulting in unstable hepatic homeostasis and increasing the risk of liver cancer (HCC/ICC). Because it is impractical to obtain experimental data on vagal nerve severance in humans, we found that non-*H. pylori* (HP)-infected perforated peptic ulcer (PPU) patients who underwent simple suture (i.e., integrated vagal nerve) or truncal vagotomy and pyloroplasty (TVP) (i.e., vagal nerve severance) could be used to study the association between the hepatic vagal system and liver cancer.

## Method

### Data source

We used an inpatient database that was a part of the Taiwan National Health Insurance Database (TNHIRD) for this retrospective cohort study. TNHIRD was established by the Taiwan National Health Insurance Administration Ministry of Health and Welfare and covers over 99% of residents in Taiwan. Inhabitants of Taiwan are obligated to join this health program. People can be lost to follow-up due to emigration, imprisonment, joining the army, or death. Because people might withdraw from this program, study subjects were followed up until the date of withdrawal or the end of 2013. The quality and accuracy of this database have been reported previously^16–18^. In addition, the International Classification of Disease, Ninth Revision, Clinical Modification (ICD-9-CM) was used to identify diseases and treatment procedures in TNHIRD. The inpatient database included all inpatient claims (containing disease diagnosis and surgery) of each insurant from 1996 to 2013. To comply with research ethics and the Personal Information Protection Act, all identifiers of insured people were shuffled and substituted with surrogate numbers for this research. This study was approved, and the need for informed consent was waived from all participants by the Research Ethics Committee of the China Medical University and Hospital in Taiwan [CMUH104-REC2-115 (CR-3)].

### Study subjects

**Figure 1:**
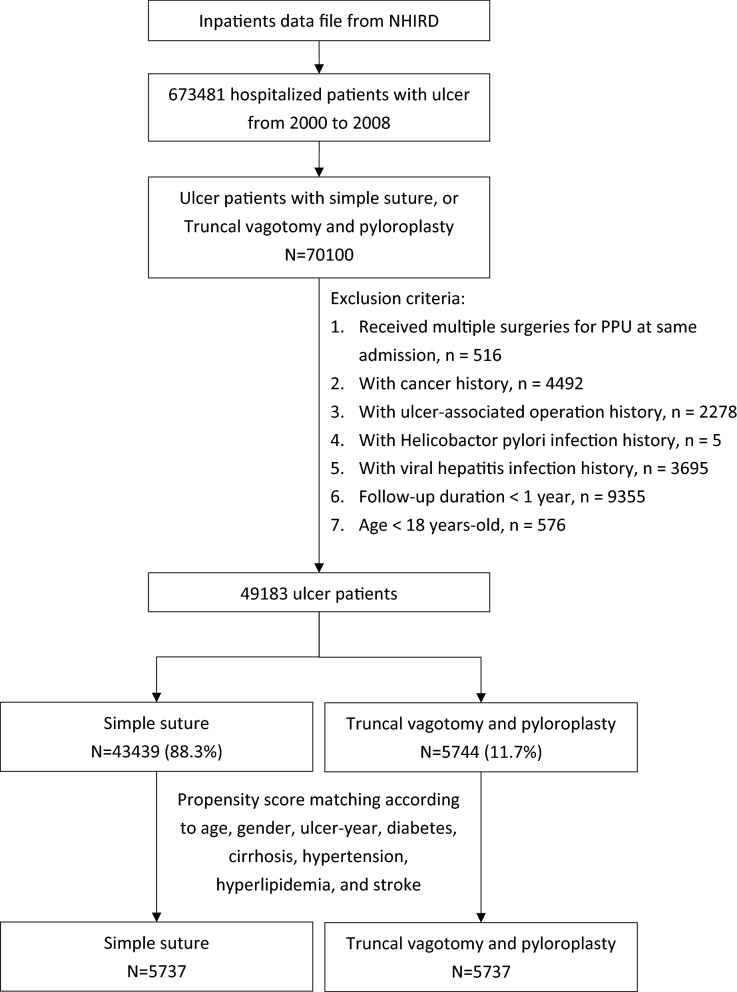
Flow chart for selecting study cohorts.

Patients with ulcer-associated surgery [including simple suture (ICD-9-CM 44.4) and truncal vagotomy/pyloroplasty (TVP, ICD-9-CM 44.01 and 44.4)] at ulcer admission (ICD-9-CM 531-533) were enrolled from 2000 to 2008. HP infection was reported to be involved in liver diseases and has a close relationship with hepatocellular carcinoma^19–22^. To avoid confounding effects, PPU patients with HP infection were excluded from this study. Therefore, the exclusion criteria included: 1. Multiple surgeries for PPU were received at the same admission; 2. Any cancer history; 3. Previous peptic ulcer-associated surgery included truncal vagotomy, suture, gastrectomy, and truncal/highly selective vagotomy; 4. HP infection history; 5. Viral hepatitis infection history; 6. Follow-up duration < one year for limiting the effect of reverse causation; and 7. Age < 18 years. As a result, there were two groups of patients: simple sutures and TVPs. To balance the baseline condition between the two groups, propensity score matching was used to select study subjects. The propensity score was estimated by logistic regression based on age, sex, ulcer admission-year, and baseline comorbidities (including diabetes, cirrhosis, hypertension, hyperlipidemia, and stroke). The matched ratio was 1:1 (Fig. 1). The propensity score matching method was based on a greedy algorithm. Matches were first made within a caliper width of 0.0000001, and the caliper width was then increased to 0.1 for unmatched cases.

### Confounder assessment and outcome

For all study participants, information on comorbidities prior to the index date was obtained, and the following comorbidities were considered: diabetes, cirrhosis, hypertension, hyperlipidemia, and stroke. These comorbidities were considered because there is an increased risk of cancer in diabetes^23^, cirrhosis^24^, hypertension^25^, hyperlipidemia^25^, and stroke^26^. These comorbidities were evaluated in the comparisons and the study cohort. The anticipated outcome was liver cancer (HCC/ICC) development (ICD-9-CM 155).

### Diagnosis and follow up of liver cancer

In addition to the ICD-9-CM system, cancer patients were diagnosed by specialists based on pathology and clinical reports and then entered into a registry system of “catastrophic illness patients” in Taiwan. Thus, HCC and ICC patients were identified from this “catastrophic illness patients” registry. All study subjects were followed from ulcer admission to the date of liver cancer development, the date of withdrawal from the database, or December 31, 2013, whichever came first.

### Statistical analysis

The distribution of categorical variables (including sex, age group, and comorbidity) is presented as numbers and percentages, and the distribution of age is presented as the mean and standard deviation (SD). The standardized difference was used to test the differences in categorical variables and continuous variables between patients with simple sutures and TVPs. The variable with the value of standardized difference > 0.1 was considered a significant difference between two cohorts. The incidence of liver cancer was calculated between groups as the sum of liver cancer development divided by the sum of follow-up duration (person-years).

Cox proportional hazard regression was used to estimate the hazard ratio and 95% confidence interval (CI) of liver cancer, and the multivariable model was adjusted for age, sex, and all comorbidities (including diabetes, cirrhosis, hypertension, hyperlipidemia, and stroke). In propensity score-matched subjects, the Cox model was used after adjusting for matched pairs. Sex-, age-, and comorbidity-stratified analyses were also evaluated. Kaplan–Meier analysis was used to plot the cumulative incidence of liver cancer, and the log-rank test was used to test the differences between the two groups. All statistical tests were two-sided, and statistical significance was defined as a p-value < 0.05. All analyses were performed using SAS, version 9.4 (SAS Institute, Cary, North Carolina.). https://www.sas.com/zh_tw/home.html.

In addition, all study methods were carried out in accordance with relevant guidelines and regulations.

## Ethics approval and consent to participate

This study was approved by the Research Ethics Committee at China Medical University and Hospital. The need for informed consent was waived from all participants [CMUH104-REC2-115 (CR-3)]. To comply with research ethics and the Personal Information Protection Act, the identifications of all insured people were shuffled and replaced with surrogate numbers for research.

## Result

### Before propensity score matching

In total, 70,100 patients who underwent ulcer-associated surgery were enrolled. After exclusion, there were 43,439 ulcer patients with simple suture treatment (88.3%) and 5,744 ulcer patients with TVP treatment (11.7%). Compared to simple suture patients, the TVP group was younger (mean age 50.2 ± 16.6 vs. 60.9 ± 16.6 years old) and was more likely to be male (82.6% vs. 69.7%) (Table 1). Simple suture patients had more comorbidities than TVP patients, specifically hyperlipidemia (7.45% vs. 1.31%).

**Table 1:** Demographics in non-HP infected PPU patients with simple suture and TVP.

	Simple suture N = 43,439		TVP N = 5744		Standardized difference	Propensity score matching				Standardized difference
						Simple suture N = 5737		TVP N = 5737		
	n	%	n	%		n	%	n	%	
**Gender**										
Women	13,165	30.3	997	17.4	0.360	1054	18.4	997	17.4	0.006
Men	30,274	69.7	4747	82.6	0.360	4683	81.6	4740	82.6	0.006
**Age, years**										
18–44	8111	18.7	2398	41.8	0.519	2369	41.3	2391	41.7	0.008
45–64	15,139	34.9	2062	35.9	0.022	1963	34.2	2062	35.9	0.036
65+	20,189	46.5	1284	22.4	0.525	1405	24.5	1284	22.4	0.050
Mean (SD)	60.9	(16.6)	50.2	(16.6)	0.644	50.4	(17.6)	50.2	(16.6)	0.008
**Comorbidity**										
Diabetes	9388	21.6	436	7.59	0.405	466	8.12	436	7.60	0.019
Cirrhosis	5622	12.9	222	3.86	0.332	190	3.31	222	3.87	0.030
Hypertension	14,701	33.8	583	10.2	0.597	618	10.8	583	10.2	0.020
Hyperlipidemia	3235	7.45	75	1.31	0.304	91	1.59	75	1.31	0.023
Stroke	5890	13.6	209	3.64	0.360	216	3.77	209	3.64	0.006

*SD* standard deviation, *HP* helicobacter pylori, *PPU* perforated peptic ulcer, *TVP* truncal vagotomy and pyloroplasty.

**Figure 2:**
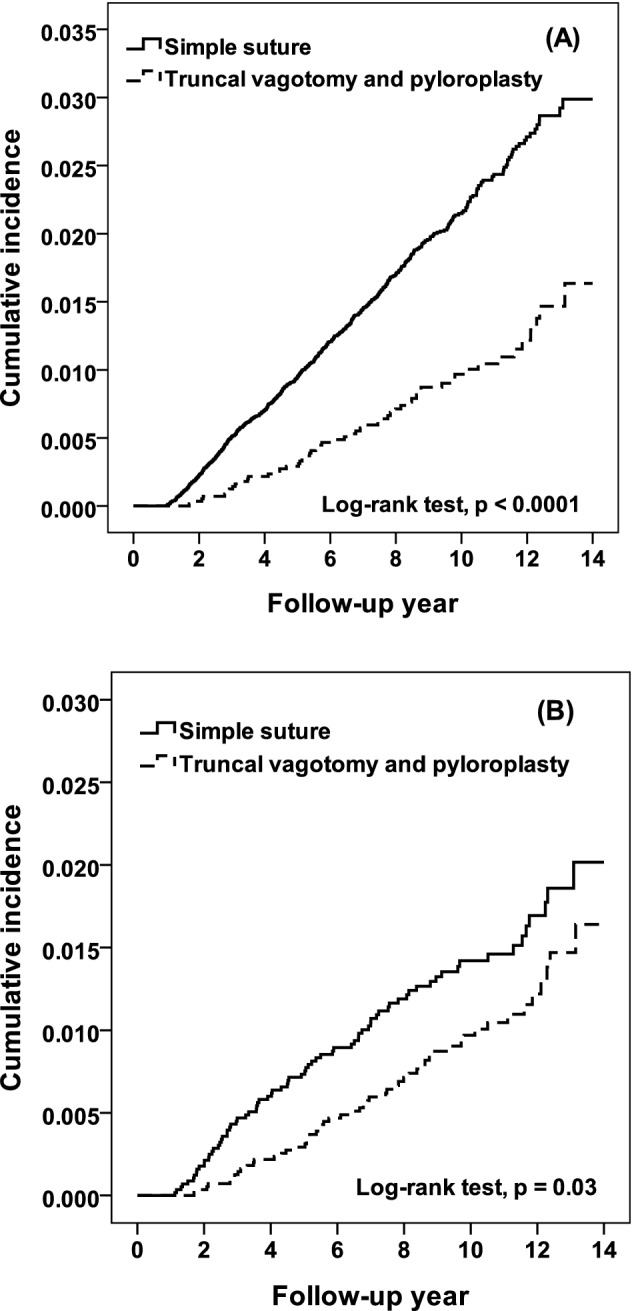
Cumulative incidence for liver cancer in non-HP infected PPU patients with simple suture and truncal vagotomy/pyloroplasty using Kaplan–Meier analysis. (**A**) All subjects, and (**B**) propensity score matching subjects.

During follow-up, there were 675 simple suture patients, and 54 TVP patients had live cancer (Table 2). This corresponds to incidences of 2.11 and 0.88 per 1000 person-years, respectively. In the multivariable Cox model, TVP patients had a 0.71-fold higher risk of liver cancer than simple suture patients (95% CI 0.54–0.95). In the stratified analysis, except in the 20- to 44-year-old age group, TVP patients were more likely to have a lower risk of liver cancer than simple suture patients, but the difference was not significant. This may be due to few events in TVP patients. However, the cumulative incidence of liver cancer in TVP patients was lower than that in simple suture patients at the 14-year follow-up (1.64% vs. 2.99%, log-rank test *p* < 0.0001) (Fig. 2A).

**Table 2:** Risk of subsequent liver and intrahepatic cancer in non-HP infected PPU patients with simple suture and TVP.

	Simple suture		TVP		TVP versus simple suture	Propensity score matching				TVP versus simple suture
						Simple suture		TVP		
	Case	Rate	Case	Rate	HR (95% CI)†	Case	Rate	Case	Rate	HR (95% CI)
Overall	675	2.11	54	0.99	0.71 (0.54–0.95)*	77	1.45	54	1.00	0.69 (0.49–0.97)*
**Gender**										
Women	168	1.79	6	0.65	0.53 (0.23–1.21)	7	0.74	6	0.65	0.88 (0.30–2.63)
Men	507	2.25	48	1.06	0.74 (0.55–1.01)	70	1.60	48	1.07	0.67 (0.46–0.96)*
**Age, years**										
20–44	48	0.68	12	0.49	1.14 (0.58–2.25)	10	0.42	12	0.50	1.19 (0.51–2.74)
45–64	278	2.29	24	1.20	0.68 (0.44–1.04)	38	2.04	24	1.20	0.59 (0.35–0.98)*
65+	349	2.73	18	1.80	0.70 (0.43–1.11)	29	2.75	18	1.80	0.66 (0.36–1.18)
**Comorbidity**										
No	205	1.23	32	0.70	0.71 (0.49–1.04)	54	1.21	32	0.70	0.57 (0.37–0.89)*
Yes	470	3.09	22	2.58	0.78 (0.51–1.20)	23	2.62	22	2.58	0.99 (0.55–1.78)

Rate, per 1000 person-years.

*HR* hazard ratio, *CI* confidence interval, *HP* helicobacter pylori, *PPU* perforated peptic ulcer, *TVP* truncal vagotomy and pyloroplasty.

^†^Multivariable model, adjusted for age, gender, diabetes, cirrhosis, hypertension, hyperlipidemia, and stroke.

*p < 0.05.

### After propensity score matching

There were 5737 simple suture patients and 5737 TVP patients enrolled. No significance in the distribution of age, sex, or comorbidities was noted (Table 1). During the follow-up duration, 145 simple suture patients and 54 TVP patients had liver cancer (Table 2). This corresponds to incidences of 1.45 and 0.88 per 1000 person-years, respectively. In the Cox model, TVP patients had a 0.69-fold higher risk of liver cancer than simple suture patients (95% CI 0.49–0.97). The cumulative incidence of liver cancer in TVP patients was lower than that in simple suture patients at the 14-year follow-up (1.64% vs. 2.02%, log-rank test *p* = 0.03) (Fig. 2b).

## Discussion

At the first encounter, there was a decreased risk of developing liver cancer (HCC/ICC) in PPU patients who underwent TVP compared to those who underwent simple closure. After stratification by sex, age, and comorbidities, there were 71% and 69% decreased risks in the TVP cohort (i.e., vagal nerve severance) compared with the simple closure cohort (i.e., integrated vagal nerve) without and with propensity score matching, respectively (Table 2). These results indicate that there might be a protective effect of TVP on the risk of liver cancer development in non-HP-infected PPU patients.

The vagus nerve plays an important role in the regulation of inflammation and metabolic homeostasis^27,28^. Persistent vagal hyperactivity is associated with increased systemic inflammatory status and systemic diseases, such as peptic ulcers^9–11^. In addition, several cohort studies reported that peptic ulcer patients have higher incidences of inflammatory diseases, such as type 2 diabetes^29^, ischemic heart disease^30^, ischemic stroke^31,32^ and liver cirrhosis^33^. In the current series, we assume that the protective effect of TVP for decreased risk of liver cancer development in non-HP-infected PPU patients might be partly caused by the relief of persistent vagal hyperactivity.

One possible explanation for this result might be attributed to the role of hepatic stellate cells (HSCs) in the human liver. HSCs play an important role in liver development, regeneration and fibrotic change^34,35^; they are in the space of Disse between hepatic sinusoidal endothelial cells and epithelial cells, with a nucleus-to-nucleus distance of 40 µm^36^, producing contraction and relaxation of the hepatic sinusoidal walls^37^. Notably, the intrahepatic efferent nerves terminate at Disse’s space, close to HSCs and hepatocytes in humans^38^. This indicates an essential role of the vagus nerve in the activation of HSCs.

HSCs play an essential role in the development of liver cancer (HCC/ICC)^39–42^. In response to hepatic injury, quiescent HSCs are activated and undergo a mesenchymal-to-epithelial transition to transdifferentiate into hepatic progenitor cells (HPCs) and hepatocytes^43^. Under a permissive microenvironment, activated HSCs may contribute to HCC development^40^, which might occur through dysregulation of liver regeneration^44^. Activated HSCs may promote the process of fibrosis and transition into alpha-smooth muscle actin-expressing contractile myofibroblasts that result in increased vascular resistance and vascular distortion. Nonetheless, activated HSCs markedly express genes involved in fibrogenesis and fibrolysis, inflammation and apoptosis^45^.

Embryologically, hepatocytes and intrahepatic biliary ducts are both derived from hepatic diverticula that originate from the endoderm^46^, which indicates a close relationship between HCC and ICC. Moreover, data from ICC patients suggested that HSCs may be related to the progression of ICC and could be potential precursors to stromal myofibroblasts and promote the development of ICC^47^.

Numerous factors and signaling pathways are involved in the activation of HSCs, such as TGF-beta, platelet-derived growth factors (PDGF), hedgehog, Notch, and microRNAs^40^. However, the hepatic nervous system has been reported to modulate^38^ as well as activate HSCs during liver carcinogenesis^47^. The hepatic vagus nerve was reported to stimulate HSC proliferation via muscarinic receptor type 2^48^ and stimulate activation of the HPC compartment via muscarinic acetylcholine receptor type 3^49^. In addition, hepatic branch vagotomy may suppress liver regeneration in partially hepatectomized rats^50,51^. Together, the vagus nerve-mediated modulation of HSCs may play an important role in the development of HCC and ICC.

Persistent vagal hyperactivity is associated with systemic inflammation and immune paralysis^52,53^. In this study, PPU patients who underwent vagotomy had a lower hazard ratio of subsequent HCC and ICC than those who underwent simple suture (HR = 0.69, Table 2). It is likely that there was persistent vagal hyperactivity in PPU patients who underwent simple suture (i.e., integrated vagal nerve). Persistent vagal hyperactivity may stimulate HSC proliferation and activate HPC^48,49^, which promotes fibrosis and dysregulates liver regeneration, as well as lead to the progression of HCC and ICC^44^. It is plausible that vagotomy alleviates persistent vagal hyperactivity in PPU patients and results in a decreased incidence of HCC and ICC.

Concern exists whether such an association is direct or indirect. For instance, obesity is a common risk factor for peptic ulcers and nonalcoholic steatohepatitis^54^ and might be associated with the development of HCC^55,56^. Moreover, abdominal operation for PPU patients may have an impact on subsequent obesity development, indicating that such an association might only be an epiphenomenon of confounding factors (e.g., obesity). However, if surgery itself had an impact, the result should be similar either in the TVP or simple closure group. In the current study, there was a significant difference in subsequent HCC and ICC between the TVP and simple closure groups (Fig. 2).

To our knowledge, there have been no similar epidemiological studies focusing on this issue. Our results showed that non-HP-infected PPU patients receiving vagotomy were associated with a lower risk of developing HCC and ICC than those receiving simple sutures (Table 2). These results indicate that there might be a role for persistent vagal hyperactivity in the activation of HSCs and an increased risk of developing HCC and ICC. Although the mechanism is partly speculative, the clinical significance of this study may, in part, provide evidence for reappraisal of the current surgical treatment of non-HP-infected PPU patients. Further studies may focus on the role of the vagus nerve in diseases and the modulation of vagal hyperactivity.

### Limitations of the study

Our study was strengthened by available data in a large population for longitudinal assessment and subgroup analysis of HCC and ICC in hospitalized non-HP-infected PPU patients undergoing various treatment procedures. However, certain limitations exist. First, lifestyle variables, such as dietary habits, smoking, drinking, socioeconomic status, and genetic information, were not obtainable for adjusting the risks of developing HCC and ICC. Second, all data used were anonymous, and there was a lack of relevant clinical variables, such as pathology findings, imaging results and laboratory data. Third, the diagnoses of patients with HCC and ICC were considered accurate based on double-checked ICD-9 codes and the “catastrophic illness patients registry” in Taiwan; however, there was a limited ability to distinguish between HCC and ICC as well as limited sample size. Fourth, biases related to retrospective studies and the limited number of patients should be noted. Because the study cohorts were well matched by sex, age, and comorbidities, the biases were likely to be minimal. The diagnosis regarding cancer diagnosis was highly reliable.

## Conclusion

Our results reported that non-HP-infected PPU patients who underwent TVP are associated with a decreased risk of subsequent HCC and ICC, which might be partly attributed to the alleviation of persistent vagal hyperactivity. However, further studies are warranted.

## Data Availability

This study used inpatient claims data from the Taiwan National Health Insurance Research Database (NHIRD). This database contains detailed medical histories of the hospitalized enrollees in Taiwan. Based on the guideline of Taiwan Ministry of Health and Welfare (TMHW), only citizens of the Taiwan are eligible to apply the NHIRD for research projects (https://nhird.nhri.org.tw/en/Data_Protection.html). The database we applied is only limited to our research purpose. All applicants must follow the Computer-Processed Personal Data Protection Law and related regulations of National Health Insurance Administration and NHRI (http://www.winklerpartners.com/?p=987). The ownership of NHIRD is belong to TMHW and the right to use is belong to the researchers. However, other researchers are able to request data access following the regulations of TMHW.
